# Medical Management of Peyronie’s Disease: Review of the Clinical Evidence

**DOI:** 10.3390/medsci7090096

**Published:** 2019-09-18

**Authors:** Patrick Teloken, Darren Katz

**Affiliations:** 1 Department of Urology, Royal Brisbane and Women’s Hospital, Brisbane, QLD 4029, Australia; 2 Department of Urology, Western Health, Melbourne, VIC 3021, Australia; dkatz@menshealthmelbourne.com.au; 3 Men’s Health Melbourne, Melbourne, VIC 3002, Australia

**Keywords:** Peyronie’s disease, medical management, non-surgical management, intralesional therapy, penile traction therapy, oral therapy

## Abstract

Peyronie’s disease is a condition that causes abnormal healing of the tunica albuginea, causing penile curvature. It is difficult to treat and its management is continuing to evolve. Proposed non-surgical treatments have included oral, topical, intralesional, extracorporeal shockwave, and traction therapy. The study of Peyronie’s disease is made difficult by heterogeneity in the timing of presentation, severity and characteristics of deformity, and associated complaints. Moreover, meta-analyses of studies are difficult due to inconsistencies across study endpoints and the duration of treatments. This article reviews the current clinical evidence and guideline recommendations, with a focus on an improvement in penile curvature.

## 1. Introduction

Peyronie’s disease (PD) is a condition with an uncertain etiology. In a susceptible individual, abnormal healing of the tunica albuginea after microtrauma results in excessive fibrosis. The usual course of PD has distinct clinical phases: (i) an early (acute) phase with ongoing inflammation, possibly pain, and evolving curvature, and (ii) a (chronic) phase with stable fibrosis. It is yet to be determined whether medical treatment in the early phase leads to improved long-term outcomes.

Several non-operative treatment strategies have been tested, including oral pharmacotherapy, intralesional injections, topical treatments, penile traction, and extracorporeal shockwave therapy (ESWT).

The goals of treatment need to be tailored to patient preferences and disease characteristics. The expected outcomes, risks, and burdens (including financial) of possible interventions need to be discussed with patients on an individual basis. Because patients with Peyronie’s disease commonly have associated erectile dysfunction, it is important to address such aspects concurrently to avoid incorrectly attributing the inability to have intercourse due to erectile function to the presence of curvature.

It is generally accepted that the primary endpoint in Peyronie’s disease is restoration of the ability to have satisfactory intercourse, and the degree of penile curvature is the commonly used objective measurement. Plaque size and the resolution of penile pain have also been used in studies as endpoints, but are of less importance.

The study of Peyronie’s disease is made difficult by heterogeneity in the timing of presentation, severity and characteristics of deformity, and associated complaints. Moreover, meta-analyses of studies are difficult due to inconsistencies across study endpoints and the duration of treatments.

A recent survey of sexual health specialists in Europe revealed that the most common primary treatments used in Peyronie’s disease are oral medication (65%), counselling (57%), and intralesional/local therapy (30%) [1]. The most commonly used oral medication was tadalafil (57%), followed by vitamin E (30%). Collagenase clostridium histolyticum (CCH) (34%) and calcium channel blockers (17%) were the most common intralesional therapies. More than one third of practitioners expressed dissatisfaction with currently available treatment options and almost two-thirds reported the impression that patients are dissatisfied with treatment outcomes [1].

The present article reviews the clinical evidence for the medical management of Peyronie’s disease, including intralesional therapy, mechanical therapy, ESWT, oral therapy, and topical therapy. A patient-centered approach is paramount: every individual deserves a thorough discussion regarding natural history and expected outcomes, including the possible risks and benefits of each treatment option, and personal preference plays a significant role in therapy selection.

## 2. Intralesional Therapy

Several agents have been used in intralesional therapy for Peyronie’s disease. Table 1 summarizes the current guidelines with regards to intralesional therapy. A recent systematic review concluded that the currently available clinical evidence from randomized trials only supports collagenase clostridium histolyticum (CCH) and IFNα-2b as being capable of improving penile curvature in patients with Peyronie’s disease [2,3].

### 2.1. Collagenase Clostridium Histolyticum

#### 2.1.1. Rationale for Use in Peyronie’s Disease

Collagenase type I (AUX-I) and type II (AUX-II) work synergistically by cleaving tropocollagen from different sites within the collagen fibrils, causing the enzymatic degradation of interstitial type I and III collagen and disruption of the plaque, while preserving the type IV collagen present in neurovascular tissue.

#### 2.1.2. Scientific Evidence

The best evidence of improvement of curvature in Peyronie’s disease exists for collagenase clostridium histolyticum. Intralesional collagenase clostridium histolyticum has been assessed in two randomized trials (IMPRESS I & II), including a total of 832 patients [8,9]. Patients with a stable dorsal curvature between 30° and 90° without complex deformity or calcification were included. Each participant underwent up to four treatment cycles (each cycle consisting of two injections 24–72 h apart of either CCH 0.58 mg or a placebo with 6 weeks of penile modeling exercises). After the second injection of each cycle, patients underwent penile plaque modeling performed by the investigator, after which they were then instructed to perform home penile modeling three times per day during the 6-week period in between each treatment cycle. The total treatment duration was 24 weeks. Collagenase led to a 34% (−17.0 ± 14.8°) improvement in penile curvature, compared to 18% (−9.3 ± 13.6°) in the placebo group. A subgroup analysis confirmed that CCH was effective in patients with curvatures of 30–60° and 61–90° [9]. 

A randomized study of 30 patients assessed whether manual modeling delivered additional benefit to patients undergoing treatment with CCH and vacuum therapy [10]. Patients received two CCH injections, as per the IMPRESS protocol, and used the vacuum device twice a day from 14 days after the second injection until the following injection. In the group randomized to modeling, this was performed 1–3 days after the second injection in each cycle. After 36 weeks, the mean reduction in curvature was 23° in both groups, suggesting that modeling brought no additional benefit.

Adverse events related to CCH include significant injection site pain, and penile swelling and hematoma. In the 551 patients randomized to CCH in the IMPRESS trials, there were three cases of corporal rupture (penile fracture) requiring surgical exploration. There were also three severe hematomas, two of which required intervention. Reported rates of corporeal rupture in clinical practice are <1% [11,12], and in a survey of sexual medicine practitioners, 34% reported having encountered at least one case of rupture [13].

Small case series suggest that collagenase clostridium histolyticum is also effective in patients with acute phase disease [11,14]. While in one of these series, patients in the acute and chronic phases of Peyronie’s achieved a similar improvement in curvature (approximately 15°), the other patients in the acute phase benefited more (20° versus 13.9°). Plaque calcification is associated with a decreased response to CCH, even if it does not impede intralesional injection. A subset analysis of the IMPRESS trials showed that while patients with no calcification and non-contiguous stippling achieved a mean reduction in curvature of approximately 17°, those with contiguous stippling (not interfering with injection) achieved a 14° reduction [9]. Moreover, in a case series of 115 patients treated with CHH, mean curvature improvement was 28% in patients without calcification and 10% in those with severe calcification [15]. Despite the poorer results, calcification does not represent a contraindication for CCH treatment, but patients need to be informed about the expected outcomes. While patients with ventral plaques were excluded from the IMPRESS trial due to concerns about urethral injury during modeling, small case series have reported at least comparable success, with no increase in adverse events [16,17]. 

CCH is an expensive and time-consuming treatment due to the cost of the medication and the number of visits required for both injections and modeling. In an attempt to reduce the costs and length of treatment, a new protocol of up to three injections, four weeks apart, with patient modeling and a vacuum pump and no practitioner remodeling, has been proposed [18]. Table 2 compares the original protocol used in the IMPRESS trials to the new proposed protocol. In a series of 53 patients with a mean curvature at baseline of 54°, this shortened protocol achieved similar results to the original protocol, with a mean improvement of 17° (31%) after three injections. Two patients developed a hematoma, one of which required aspiration. There were no cases of corporal rupture.

### 2.2. Intralesional Interferon α-2b

#### 2.2.1. Rationale for Use in Peyronie’s Disease

Intralesional interferon α-2b has resulted in alteration of the metabolic activity of myofibroblasts and decreased fibroblast and collagen proliferation, with an increase in collagenase production.

#### 2.2.2. Scientific Evidence

Intralesional interferon α-2b has been shown to be superior to a placebo in improving penile curvature and plaque size in two small placebo-controlled studies [19,20]. Both studies had a mean pre-treatment curvature of approximately 50°. Curvature improvement was approximately 12 and 13.5° in the interferon arms and 3.6 and 4.3° in the placebo arms in both studies. Despite its efficacy, treatment side-effects, which include myalgias, arthralgia, sinusitis, fever, and flu-like symptoms, as well as its cost, mean that it is rarely used.

### 2.3. Verapamil

#### 2.3.1. Rationale for Use in Peyronie’s Disease

Verapamil remodels and degrades extracellular fibrosis by (1) inhibiting the synthesis/secretion of extracellular matrix molecules, including collagen, glycosaminoglycans, and fibronectin; (2) increasing collagenase activity; and (3) modifying transforming growth factor-ß (TGF-ß) activity.

#### 2.3.2. Scientific Evidence

Conflicting results have been reported by the four comparative randomized studies assessing the role of intralesional verapamil injection that have been reported to date. Only one of these studies compared verapamil to a placebo, and included a total of 14 patients [21]. The mean reduction in curvature was 8° with verapamil and 2° with a placebo. A randomized study of intralesional verapamil versus hyaluronic acid showed that after 12 weeks of weekly treatment, there was no change in curvature in the verapamil group and a reduction of 4.6° in the hyaluronic acid group [22].

Of note, a small study randomized patients with a mean curvature of 28° to receive 10 mg verapamil diluted in 4 mL, 10 mL, or 20 mL of saline. Injections were performed every 2 weeks for a total of 12 treatments. The mean change in curvature was 4.7°, 6.7°, and 13.6° in the 4 mL, 10 mL, and 20 mL groups, respectively, suggesting that hydrodistension of the plaque might have an effect, rather than the medication itself [23].

Currently, there is no good-quality evidence from randomized studies to support the use of verapamil. 

### 2.4. Hyaluronic Acid

#### 2.4.1. Rationale for Use in Peyronie’s Disease

Hyaluronic acid modulates many aspects of tissue repair, including the activation of inflammatory cells and fibroblast activity, potentially reducing fibrosis. 

#### 2.4.2. Scientific Evidence

A single randomized controlled study assessing hyaluronic acid has been reported [22]. Patients were randomized to hyaluronic acid or verapamil weekly for 12 weeks. There was no change in curvature in the verapamil group and a reduction of 4.6° in the hyaluronic acid group. While hyaluronic was statistically better than verapamil, the clinical significance of a mean change of 4.6° is doubtful. A series of 83 patients treated with a total of 30 hyaluronic acid 20 mg injections over 6 months was compared to 81 untreated controls. Both groups were comprised of patients with a curvature of less than 45° and that were in the acute phase of disease. After 1 year, there was a mean reduction in curvature of 9° in the treated group compared to the untreated group [24]. A study of 65 patients with a mean curvature of 30° who underwent weekly intraplaque injections of 8 mg hyaluronic acid for 10 weeks reported a mean decrease in penile curvature of 10° (37%) [25].

These studies do not provide robust evidence to allow recommendations to be made on the use of hyaluronic acid.

### 2.5. Steroids

#### 2.5.1. Rationale for Use in Peyronie’s Disease

Steroids resulted in an inhibition of inflammatory and immune responses via glucocorticoid-receptor mediated effects on gene expression.

#### 2.5.2. Scientific Evidence

Intralesional steroids have not been shown to have an advantage over a placebo in randomized studies. Due to the lack of effectiveness and potential side effects, they are currently contraindicated.

### 2.6. Plasma-Rich Platelets (PRP) and Hyaluronic Acid

In a series of 90 patients treated with 4–8 injections of a combination of PRP and hyaluronic acid every 2 weeks for the first 4 and monthly thereafter, a mean curvature improvement of 16° (39%) was reported [26]. Further randomized controlled studies are necessary to assess the efficacy of this treatment. 

### 2.7. Meta-Analysis of Intralesional Therapies

A network meta-analysis of eight randomized controlled studies suggested that in regards to penile curvature, CCH and interferon α-2b are associated with better outcomes than verapamil or hyaluronic acid [3]. 

## 3. Mechanical Therapy: Traction and Vacuum

### 3.1. Rationale for Use in Peyronie’s Disease

Traction and vacuum therapies have resulted in remodeling of the extracellular matrix of the plaque via mechanically-induced signal transduction pathways and gene regulatory mechanisms.

### 3.2. Scientific Evidence

Three small case series of penile traction and vacuum devices in patients with PD reported conflicting results, likely due to heterogeneity in the patient population and treatment protocols. In the first report, patients with stable disease used the traction device for 2–8 h per day for 6 months, and extender rods were lengthened by 0.5 cm every 2 weeks [27]. Ten patients were included and the mean curvature improvement was 17° (33%). In another study of men with stable disease, including 19 patients with a curvature up to 50°, the use of a traction device for at least 5 h a day for 6 months did not lead to a significant improvement in curvature (31° pre-treatment and 27° post-treatment) [28]. A prospective study compared the outcomes of the use of traction therapy in 55 patients with acute phase disease to those of 41 contemporary patients who declined such therapy and received oral treatment instead [29]. Baseline curvature was 33° in the traction therapy and 29° in the oral therapy group.

Patients were instructed to use the traction device for 6–9 h a day, for at least 6 months. At 9 months, there was a mean curvature reduction of 20° (60%) in the traction group, while the oral treatment group suffered a mean increase of 22° (75%). The authors reported that traction obviated the need for surgery in 40% of patients and in one of every three patients, made plication possible instead of grafting. 

Recently, two studies using novel traction devices have been reported. The Penimaster PRO was evaluated in a randomized controlled trial of 93 men with at least 45° curvature and stable disease [30]. After 12 weeks of daily use of the traction device for 3–8 h, there was a 31° (41%) improvement in the treatment group and no change in the no intervention group. A subset analysis suggested a dose-dependent response, with a longer usage time being associated with greater curvature improvement. Mild side effects, including glans numbness and local discomfort, were reported by 43% of respondents.

RestoreX is a penile traction device that uses a ratcheting body with springs to generate longitudinal and oppositional angular forces and is used for between 30 and 90 min per day. A randomized study of 110 men with stable disease and curvature greater than 30° showed that after 3 months, there was an 11° curvature reduction in the treatment group and a 1.3° increase in the control group [31]. 

Vacuum therapy was assessed in a series of 31 patients with a mean disease duration of 9.9 months [32]. The treatment protocol comprised of vacuum use 10 min twice daily for 12 weeks. The authors reported that 67% of patients exhibited an improvement in curvature of between 5 and 25°, but the overall mean improvement was 7.4° (15% mean improvement in curvature).

The role of mechanical therapies in combination with intralesional treatments has also been explored. In a non-randomized comparison of patients undergoing treatment with intralesional treatment, the use of penile traction was not associated with a greater improvement in curvature in association with verapamil [33] or interferon α-2b [34]. 

An analysis of a prospectively collected database of patients who underwent CCH intralesional therapy compared three subsets of patients: (1) traction therapy with RestoreX; (2) traction with other devices; and (3) no have traction therapy. The mean curvature improvement was 33.8° (49%), 19.2° (30%), and 20.3° (31%), respectively.

Additionally, traction therapy can have a role in preserving/increasing penile length pre- or postoperatively.

In summary, mechanical therapy is now supported by randomized controlled trials in the acute and stable phase of Peyronie’s disease to improve penile curvature. It is expected that guidelines (Table 3) will be updated and further support its use given the recent publication of two randomized controlled trials.

## 4. Extracorporeal Shockwave Therapy (ESWT)

### 4.1. Rationale for Use in Peyronie’s Disease

ESWT has been shown to cause plaque damage and, via mechanotransduction, increase tissue nitric oxide and vascular endothelial growth factor (VEGF), potentially leading to plaque resorption.

### 4.2. Scientific Evidence

Two of three randomized trials on Peyronie’s disease (PD) involving 238 patients reported an improvement in pain; however, no clinically significant changes in penile deviation and plaque size were observed [35,36,37]. Therefore, the use of ESWL to improve curvature in Peyronie’s disease is currently discouraged by guidelines (Table 4).

## 5. Oral Therapy

Several agents have been studied for the treatment of Peyronie’s disease. Table 5 summarizes the current guidelines with regards to oral therapy, and Table 6 the results from randomized controlled trials. To date, no oral treatment has proven to be effective in improving penile curvature in patients with stable disease. From a theoretical perspective, it makes sense that once a penile fibrotic plaque has formed, oral (systemic) treatments will be ineffective. It seems possible that oral (systemic) treatment could modulate healing in the acute phase and prevent deterioration/lead to improvement, but none of the medications studied to date have consistently been shown to have such an effect.

### 5.1. Phosphodiesterase Type-5 Inhibitors (PDE-5i)

#### 5.1.1. Rationale for Use in Peyronie’s Disease

PDE-5i decreases inflammatory changes associated with oxidative stress, the collagen to smooth muscle ratio, the collagen III to collagen I ratio, and the number of myofibroblasts, and selectively increases the apoptotic index.

#### 5.1.2. Scientific Evidence

There is no high-quality evidence that PDE-5 inhibitor use leads to an improvement in penile curvature. In a retrospective case-control study of 65 patients presenting to a sexual dysfunction clinic who had a septal scar on a penile ultrasound, daily low-dose tadalafil use for 6 months was associated with a greater resolution of the sonographically detected septal scar. Of note, only 11% of patients had penile deformity, with the remaining having no complaints or palpable plaque [47]. In a randomized trial comparing ESWL alone to the combination of ESWL and 5 mg tadalafil daily, no significant change in penile curvature was seen in either group [48]. It is well-established that a significant proportion of patients with Peyronie’s disease also suffer from erectile dysfunction [49,50]. Therefore, even though there is no evidence to support PDE-5 inhibitor use for curvature improvement, their use is often justified to improve erectile function. 

### 5.2. Vitamin E

#### 5.2.1. Rationale for Use in Peyronie’s Disease

Vitamin E stimulates antioxidant activity by inactivating free radicals and reducing oxidative stress.

#### 5.2.2. Scientific Evidence

A randomized, double-blind, crossover, placebo-controlled trial was published in 1983 [38]. Sixty men randomly received vitamin E (200 mg) or a placebo three times daily for 3 months each (Table 7). Patients were evaluated monthly based on the severity of symptoms (pain, deformity, quality of erection, capacity of penetration, and coitus). Only 40 patients completed the study (67%), and vitamin E was not different from the placebo, with the possible exception of the improvement of pain. Despite a lack of scientific evidence of its action in PD, vitamin E is still widely used due to being inexpensive and having minimal side effects.

### 5.3. Para-Aminobenzoate Potassium (POTABA)

#### 5.3.1. Rationale for Use in Peyronie’s Disease

POTABA use has been shown to result in stabilization of the serotonin-mono-amine oxidase activity and a direct inhibitory effect on fibroblast glycosaminoglycan secretion would have anti-inflammatory and anti-fibrotic effects.

#### 5.3.2. Scientific Evidence

A multicenter RCT with 60 men compared 12 months of treatment with 4 g POTABA three times a day and a placebo. The final report of this study was never published; however, a preliminary report of the outcome for 41 men showed no benefit of the active treatment, except for a possible improvement in pain [51]. 

A randomized trial compared the administration of 3 g POTABA four times a day to a placebo in men with PD for less than 12 months, noncalcified plaques, and without previous treatment [39]. Of 103 randomized men, 75 were included in the final analysis, as 11 stopped treatment due to side effects (13% and 7% of patients in POTABA and placebo groups, respectively) and 17 were excluded due to non-compliance. Of note, 62 of the 75 had penile curvature at the start of the study. After 12 months of therapy, no relevant difference in the improvement of pre-existing penile deviation was found (Table 8). While the authors suggested that POTABA may stabilize the disease as progression or development of curvature was less common in the POTABA group (3% vs. 21%), this was a subgroup post-hoc analysis, and thereby, is only hypothesis-generating. Moreover, the strict inclusion and exclusion criteria impede the generalization of findings to the typical Peyronie’s patient. In summary, the lack of conclusive evidence, cost, and side effects of POTABA limit its use in Peyronie’s disease.

### 5.4. Colchicine

#### 5.4.1. Rationale for Use in Peyronie’s Disease

Colchicine exerts antifibrotic, antimitotic, and anti-inflammatory activities by depolymerizing microtubules.

#### 5.4.2. Scientific Evidence

A single RCT studied 84 patients with noncalcified plaque [40]. The mean disease duration was 15 months, and patients received 0.5–2.5 mg of colchicine and a placebo. An objective evaluation of the plaque and symptoms failed to demonstrate any action of colchicine (Table 9).

### 5.5. Colchicine plus Vitamin E

#### Scientific Evidence

A single-blind study compared the use of 1 mg colchicine twice daily plus 600 mg vitamin E twice daily vs. 200 mg ibuprofen twice daily for 6 months in 45 patients with PD in the early stages (time from onset <6 months), a penile curvature of less than 30°, and no erectile dysfunction [41]. Plaque size and penile curvature significantly decreased in the group receiving colchicine plus vitamin E (Table 10). Of note, these patients do not represent typical patients presenting with Peyronie’s disease.

### 5.6. Procarbazine

#### 5.6.1. Rationale for Use in Peyronie’s Disease

Procarbazine results in the inhibition of fibroblast proliferation.

#### 5.6.2. Scientific Evidence

In an open-label crossover study, 34 men were randomly assigned to receive procarbazine (20 mg twice daily) or vitamin E (200 mg three times daily) for 3 months and after the other drug [42]. Vitamin E was superior to procarbazine in regards to the improvement in curvature (Table 11). Moreover, only 67% of patients completed the study. Given the lack of evidence to support its use and side effects, procarbazine use is discouraged.

### 5.7. Tamoxifen

#### 5.7.1. Rationale for Use in Peyronie’s Disease

Tamoxifen results in the inhibition of keloid fibroblast proliferation and collagen production by decreasing TGF-ß production.

#### 5.7.2. Scientific Evidence

A double-blinded study of 25 patients with a mean disease duration of 20 months randomized patients to tamoxifen (20 mg twice daily) or a placebo [43]. There were no differences in any of the investigated parameters (Table 12). 

### 5.8. Carnitine

#### 5.8.1. Rationale for Use in Peyronie’s Disease

Carnitine causes the inhibition of acetyl-coenzyme A, an increase in mitochondrial activity, and a decrease in free radicals.

#### 5.8.2. Scientific Evidence

The action of acetyl-L-carnitine was assessed in two RCTs. In the preliminary report, 48 patients (15 “acute” phase and 33 “chronic” phase) were randomly assigned to 20 mg tamoxifen twice daily or 1 g acetyl-L-carnitine twice daily for 3 months [44]. At 6 months, acetyl-L-carnitine led to a modest improvement in penile curvature not seen in the tamoxifen group (Table 13). The plaque size decreased in both groups. Tamoxifen induced significantly more side effects than acetyl-L-carnitine. Of note, patients in this study do not represent typical patients with PD, because they had a mild severity of curvature, and the mean duration of disease before seeking medical treatment was only 5 weeks.

In a second study utilizing propionyl-L-carnitine, 60 men were randomly assigned in a double-blind design to receive propionyl-L-carnitine (1 mg twice daily) or tamoxifen (20 mg twice daily) for 3 months [45]. All patients received weekly intraplaque verapamil (10 mg) injections. The group receiving carnitine exhibited a significantly greater improvement in curvature (11.8° vs. 1.9°; Table 14).

### 5.9. Pentoxifylline

#### 5.9.1. Rationale for Use in Peyronie’s Disease

Pentoxifylline decreases fibroblast proliferation and collagen and elastin deposition while increasing fibroblast apoptosis and fibrinolysis via the inhibition of TGF-ß, the fibroblast growth factor (FGF), and the platelet-derived growth factor (PDGF).

#### 5.9.2. Scientific Evidence

The only randomized placebo-controlled trial of pentoxifylline has been retracted due to “statistical inconsistencies” [46].

## 6. Topical Therapy

A double-blind randomized trial suggested that 15%verapamil gel twice daily for 3 months led to a 43% mean reduction in curvature compared to 18% in the placebo group [52]. Of note, curvature severity was measured based on the patient’s subjective assessment, there were only 18 patients in each group, and there was great heterogeneity in this sample with regards to disease duration (2 months to 15 years, mean 3 years) and curvature severity (5° to 120°, mean 45°).

Concerns about the ability of topical verapamil preparations to penetrate the tunica albuginea led to the exploration of transdermal electromotive drug administration (EDMA). A randomized trial of 96 men compared the administration of 5 mg verapamil and 8 mg dexamethasone (treatment) or lignocaine (control) four times a week for 6 weeks [53]. When evaluating the 73 (76%) patients who completed the study, median penile curvature decreased from 43 to 21° in the treatment group and remained stable at 41° in the control group. Another randomized study of 42 men found no benefit of a twice weekly regime of 10 mg verapamil for 3 months over a placebo (mean curvature improvement 9 vs. 7°) [54]. Further studies are needed to clarify these inconsistent results.

A new topical treatment called H-100, containing nifedipine and superoxide dismutase, using emu oil as a carrier agent, has been assessed in a randomized trial of 22 patients with acute phase Peyronie’s disease and a mean curvature of approximately 50° [55]. Gel applications were conducted twice daily. One group received a placebo for 3 months, followed by H-100 for 3 months, while the second group received H-100 for the entire 6 months. At 3 months, mean curvature reductions of 1.2° (2.5%) and 13.9° (27%) were reported for the placebo and H-100 groups, respectively. At 6 months, the mean improvement in curvature was 17° (37%) in patients who had used H-100 for 3 months and 20° (40%) in patients who had been using H-100 for 6 months. A self-limiting skin rash was the only reported side-effect. These findings need to be replicated before recommendations can be made.

None of the current guidelines recommend topical therapy in Peyronie’s disease (Table 15). While the EAU guidelines state that “topical verapamil gel 15% may improve penile curvature”, the only published study did not have an objective measurement of curvature, as well as other methodological shortcomings, as discussed above.

## 7. New Developments

Stem-cell therapy is an exciting field that might have a role in the management of Peyronie’s disease in the future. While animal studies have reported promising results, clinical studies are still awaited [56].

## 8. Conclusions

A great number of medical treatments have been suggested and utilized in PD. Evidence from randomized controlled trial with regards to improvement in curvature exists for intralesional (with CCH and interferon α-2b) and traction therapy. Oral therapy has not proven to be effective and due to potential side-effects and costs, is not currently recommended. Topical verapamil with or without EMDA is not recommended and novel topical agents need further studies prior to routine use.

An optimal treatment algorithm applying a multimodal approach is yet to be defined. With advances in the comprehension of the mechanisms of inflammation and scarring and the development of new agents, it is expected that more effective treatments for PD will become available.

## Figures and Tables

**Table 1:** Guidelines on Intralesional Therapy for Peyronie’s Disease.

ISSM (2016) [4]	Some outcome benefits of CCH, interferon, and verapamil
AUA (2015) [5]	Clinicians may administer CCH, interferon, or verapamil
CAU (2018) [6]	CCH as first-line therapy, with the use of verapamil or interferon as a second-line option
EAU (2019) [7]	Intralesional treatment with CCH showed significant decreases in the deviation angle, plaque width, and plaque length. Intralesional treatment with interferon may improve penile curvature, plaque size and density, and pain. Intralesional treatment with steroids is not associated with a significant reduction in penile curvature, plaque size, or penile pain. Do not use intralesional treatment with steroids to reduce penile curvature, plaque size, or pain.

ISSM: International Society for Sexual Medicine; AUA: American Urological Association; CAU: Canadian Association of Urology; EAU: European Association of Urology; CCH: collagenase clostridium histolyticum.

**Table 2:** Comparison of Protocols for CCH use.

	IMPRESS Protocol	Modified Shortened Protocol
Cycle description	Two CCH injections, 2–3 days apart Practitioner modeling 2–3 days after 2nd injection Patient modeling Repeat after 6 weeks, for up to 4 cycles	One CCH injection Patient modeling and vacuum pump use Repeat after 4 weeks, for up to 3 cycles
Number of injections	8	3
Total number of visits (including first assessment visit)	14	4
Duration of treatment	24 weeks	12 weeks
Number of patients	551	53
Curvature improvement	17° (34%)	17° (31%)

**Table 3:** Guidelines on Traction for Peyronie’s Disease.

ISSM (2016)	The use of penile traction therapy could have some benefits in PD.
AUA (2015)	-
CAU (2018)	Recommended based on low-level evidence.
EAU (2019)	Use penile traction devices and vacuum devices to reduce penile deformity and increase penile length.

ISSM: International Society for Sexual Medicine; AUA: American Urological Association; CAU: Canadian Association of Urology; EAU: European Association of Urology.

**Table 4:** Guidelines on Extracorporeal Shockwave Therapy (ESWL) for Peyronie’s Disease.

ISSM (2016)	Minimal impact on correcting deformity, but provides a more rapid decrease of pain and stabilization of curvature in patients with PD.
AUA (2015)	Clinicians should not use extracorporeal shock wave therapy (ESWT) for the reduction of penile curvature or plaque size.
CAU (2018)	Not recommended for the reduction of curvature or plaque size.
EAU (2019)	Extracorporeal shockwave treatment does not improve penile curvature and plaque size, but it may be offered for penile pain

**Table 5:** Guidelines on Oral Therapy for Peyronie’s Disease.

ISSM (2016)	Minimal or no benefit with respect to a significant decrease in deformity with any oral therapy.
AUA (2015)	NSAIDs may be used for pain. Clinicians should not offer oral therapy with vitamin E, tamoxifen, procarbazine, omega-3 fatty acids, or a combination of vitamin E with L-carnitine.
CAU (2018)	No proven efficacy/limited potential efficacy and may have deleterious side effects.
EAU (2019)	Do not use oral treatment with vitamin E and tamoxifen for a significant reduction in penile curvature or plaque size. Do not offer other oral treatments (acetyl esters of carnitine, pentoxifylline, colchicine) for the treatment of PD.

ISSM: International Society for Sexual Medicine; AUA: American Urological Association; CAU: Canadian Association of Urology; EAU: European Association of Urology.

**Table 6:** Summary of findings from oral therapy randomized controlled trials on curvature.

		*N*	Curvature Improvement
**PDE-5i**	No RCTs		
**Vitamin E** [38]	RCT, crossover	60	Vit E: 7.8% Placebo 0%
**POTABA** [39]	RCT	62	POTABA: 63% Placebo: 59%
**Colchicine** [40]	RCT	84	Colchicine: 17% Placebo 18%
**Vitamin E + Colchicine** [41]	RCT	45	Vit E + Colchicine: 46% Ibuprofen: 18%
**Procarbazine** [42]	RCT, crossover	34	Procarbazine: 9% Vit E: 37%
**Tamoxifen** [43]	RCT	25	Tamoxifen: 46% Placebo: 425
**Carnitine** [44]	RCT	48	Curvature change Carnitine: −7.5° Tamoxifen: −0.5°
**Carnitine + Intralesional Verapamil** [45]	RCT	60	Curvature change Carnitine + Verapamil: −11.8° Verapamil: −1.9°
**Pentoxifylline** [46]	RCT retracted		

RCT: randomized controlled trial.

**Table 7:** Vitamin E vs. Placebo: Randomized, Double-Blind Crossover Study [38].

	*N*	200 mg Vitamin E Three Times Daily, 3 Months	Placebo
Pain improvement	14	5 (35.7%)	1 (7.1%)
Curvature improvement	38	3 (7.89%)	0
Ability to have sex	35	5 (14.2%)	3 (8.57%)

**Table 8:** POTABA use in acute Peyronie’s disease: Randomized, Double-Blind, Placebo-Controlled.

	3 g POTABA Four Times a Day, 12 Months *N* = 30	Placebo, 12 Months *N* = 32
Curvature		
Improvement	19 (63%)	19 (59%)
No change	10 (33%)	8 (18%)
Increase	1 (3%)	7 (21%)

**Table 9:** Colchicine vs. Placebo: Randomized, Double-Blind, Placebo-Controlled.

	0.5–2.5 mg	
	Colchicine Daily,	Placebo,
	4 Months	4 Months
	Improvement	
Pain	60%	63.6%
Curvature change	17.1%	18.4%
Plaque size	10.5%	10%

**Table 10:** Vitamin E plus Colchicine vs. Ibuprofen: Randomized, Single-Blind.

	300 mg Vit E Twice Daily Plus 1 g Colchicine Twice Daily, 6 Months	200 mg Ibuprofen Twice Daily, 6 Months
	Improvement		
Pain	21 (91%)	15 (68%)
Curvature change	6 (46%)	4 (18%)
Plaque size change (cm)	−0.26	+0.13

**Table 11:** Procarbazine vs. Vitamin E: Randomized, Crossover Study.

	50 mg Procarbazine	200 mg Vitamin E
	Twice Daily, 3 Months	Three Times Daily, 3 Months
Curvature improvement	9%	37%
Curvature resolution	0	6.45%
No change	86.3%	61.2%
Curvature worsening	4.5%	0

**Table 12:** Tamoxifen vs. Placebo: Randomized, Double-Blind, Placebo-Controlled.

	*N*	Tamoxifen 20 mg	*N*	Placebo
		Twice Daily, 3 Months		Twice Daily, 3 Months
		Improvement		Improvement
Pain	6	4 (66%)	4	3 (75%)
Curvature	13	6 (46%)	12	5 (42%)
Plaque size	13	4 (31%)	12	3 (25%)

**Table 13:** Carnitine vs. Tamoxifen: Randomized, Double-Blind.

	Carnitine 1 g Twice Daily, 3 Months	20 mg Tamoxifen, 3 Months
Pain improvement	22 (92%)	15 (68%)
Curvature change	−7.5°	−0.5°
Plaque size change (mm^2^)	48.8	26.9

**Table 14:** Carnitine vs. Tamoxifen: Randomized, Double-Blind.

	Carnitine 1g oral Twice Daily plus Verapamil 10mg Intraplaque Weekly, 3 Months	Tamoxifen 20mg oral twice Daily plus Verapamil 10 mg Intraplaque Weekly, 3 Months
Curvature change	11.8° (30%)	1.9° (5%)
Plaque size change (mm2)	7.6	1.3

**Table 15:** Guidelines on Topical Therapy for Peyronie’s Disease.

ISSM (2016)	The use of topical verapamil and iontophoresis is not recommended in PD.
AUA (2015)	Clinicians should not offer electromotive therapy with verapamil.
CAU (2018)	Iontophoresis: Not recommended. Absence of convincing efficacy and a substantial burden of administration. Verapamil gel: uncertain.
EAU (2019)	Topical verapamil gel 15% may improve penile curvature and plaque size. Iontophoresis with 5 mg verapamil and 8 mg dexamethasone may improve penile curvature and plaque size.
